# Pulmonary tuberculosis and tuberculous pleuropericarditis in a patient with pyoderma gangrenosum treated with adalimumab: a case report

**DOI:** 10.1186/s13256-026-06129-1

**Published:** 2026-05-27

**Authors:** Kei Kanata, Shoma Matsushita, Yutaro Ito, Koshiro Ichijo, Masahiro Uehara

**Affiliations:** Department of Respiratory Medicine, Shimada General Medical Center, 1200-5 Noda, Shimada City, Shizuoka Prefecture 427-8502 Japan

**Keywords:** Tuberculosis, Pleuropericarditis, Adalimumab, Case report

## Abstract

**Background:**

Adalimumab is a tumor necrosis factor-α inhibitor, which is associated with an increased risk of tuberculosis. However, evidence regarding tuberculosis development in patients with pyoderma gangrenosum is limited, and simultaneous involvement in lungs, pleura, and pericardium is rare. This report describes a case of pulmonary tuberculosis and tuberculous pleuropericarditis associated with adalimumab administration.

**Case presentation:**

The patient was a 59-year-old Filipino female. She began receiving adalimumab to treat pyoderma gangrenosum at the dermatology department of our hospital. The serum interferon-gamma release assay using QuantiFERON test, measured prior to administration, was negative. After five times of administration, she presented with fever, cough, and dyspnea. A chest computed tomography scan showed multiple small nodular shadows in the lung fields, right pleural effusion, and pericardial effusion. The administration of antibiotics and diuretics was ineffective. The QuantiFERON test was repeated, and was positive. The rapid sputum test for tuberculosis antigen was positive, and *Mycobacterium tuberculosis* was subsequently cultured 23 days later. Thus, she was diagnosed with pulmonary tuberculosis. Standard treatment with a combination of four antituberculous drugs was started, but drug susceptibility testing revealed resistance to isoniazid, so isoniazid was changed to levofloxacin. After treatment, multiple small nodular shadows in the lung fields disappeared, and the pleural and pericardial effusions completely resolved. Thus, we clinically diagnosed pulmonary tuberculosis with combined tuberculous pleuropericarditis. To the best of our knowledge, there have been no reports of pulmonary tuberculosis and tuberculous pleuropericarditis following adalimumab administration.

**Conclusion:**

This case highlights the importance of considering both pulmonary and extrapulmonary tuberculosis as potential complications of adalimumab, even in patients with initially negative interferon-gamma release assay results. Repeat testing may be crucial for early detection, especially in high-risk individuals from endemic areas.

## Background

Adalimumab is a tumor necrosis factor (TNF)-α inhibitor that was approved by the Food and Drug Administration (FDA) as the first fully human monoclonal antibody in 2002. TNF-α inhibitor is widely used in inflammatory conditions such as rheumatoid arthritis and pyoderma gangrenosum. However, TNF-α plays a critical role in maintaining granuloma integrity and controlling latent tuberculosis (TB) infection. Its inhibition increases the risk of TB reactivation. Interferon-gamma release assay (IGRA) is widely used for latent TB screening; however, false-negative results may occur, particularly in immunosuppressed patients. Therefore, repeated testing may be necessary in high-risk populations [1, 2]. In addition, evidence regarding TB risk in patients with pyoderma gangrenosum receiving anti-TNF-α therapy remains limited. Although there have been a few reports of tuberculous pleurisy or pericarditis, to the best of our knowledge, there have been no reports of pulmonary tuberculosis and tuberculous pleuropericarditis following adalimumab administration to pyoderma gangrenosum. This report describes a rare case of pulmonary tuberculosis and tuberculous pleuropericarditis following adalimumab therapy for pyoderma gangrenosum and highlights the limitations of a single baseline IGRA screening.

## Case presentation

The patient was a 59-year-old female from the Philippines who had been living in Japan for several years. She had a medical history of diabetes mellitus and hypertension, both of which had been well controlled for approximately 10 years with metformin 500 mg, sitagliptin 50 mg, dapagliflozin 10 mg, and amlodipine 5 mg. She also had pyoderma gangrenosum, for which she had been treated in the dermatology department of our hospital. There were no relevant genetic findings or family history suggestive of inherited conditions. She was started on prednisone for pyoderma gangrenosum on April 21 at a dose of 30 mg/day, then tapered to 20 mg/day on April 28 and to 10 mg/day on May 9 of a particular year.

Because the response to prednisone was ineffective, the patient was switched to adalimumab on May 9 at a dose of 160 mg, and prednisone administration was discontinued. Adalimumab was tapered to 80 mg on May 23 and 40 mg on June 13, June 20, and August 15, and prednisone administration was discontinued. The serum IGRA using QuantiFERON (QIAGEN, Hilden, Germany) test, measured prior to administration on April 21, was negative. On August 16, when it had been approximately 3 months after the initiation of adalimumab therapy, the patient presented with symptoms of fever ranging from 37 ℃ to 38 ℃, together with cough, dyspnea, and these symptoms continued for about one month. She visited the respiratory medicine department for an investigation on September 12. A physical examination revealed a body temperature of 37.7 °C, a heart rate of 122 beats per minute, and decreased breath sounds were noted in the right lung. The patient’s blood pressure was within the normal range (126/78 mmHg), and no cervical lymphadenopathy or abnormal heart murmurs were detected. Laboratory test results showed a white blood cell count of 5800 /μL (neutrophil count of 3190 /µL, lymphocyte count of 2100 /µL), C-reactive protein (CRP) of 1.57 mg/dL, and serum soluble interleukin-2 receptor (sIL-2R) of 1519 U/mL. N-terminal pro B-type natriuretic peptide (NT-proBNP) was not elevated at 102 pg/mL. β-D-Glucan, anti-mycobacterium avium complex (MAC) antibody, and human immunodeficiency virus (HIV) antibodies were negative. There was no significant increase in antibodies suggestive of collagen disease.

**Fig. 1:**
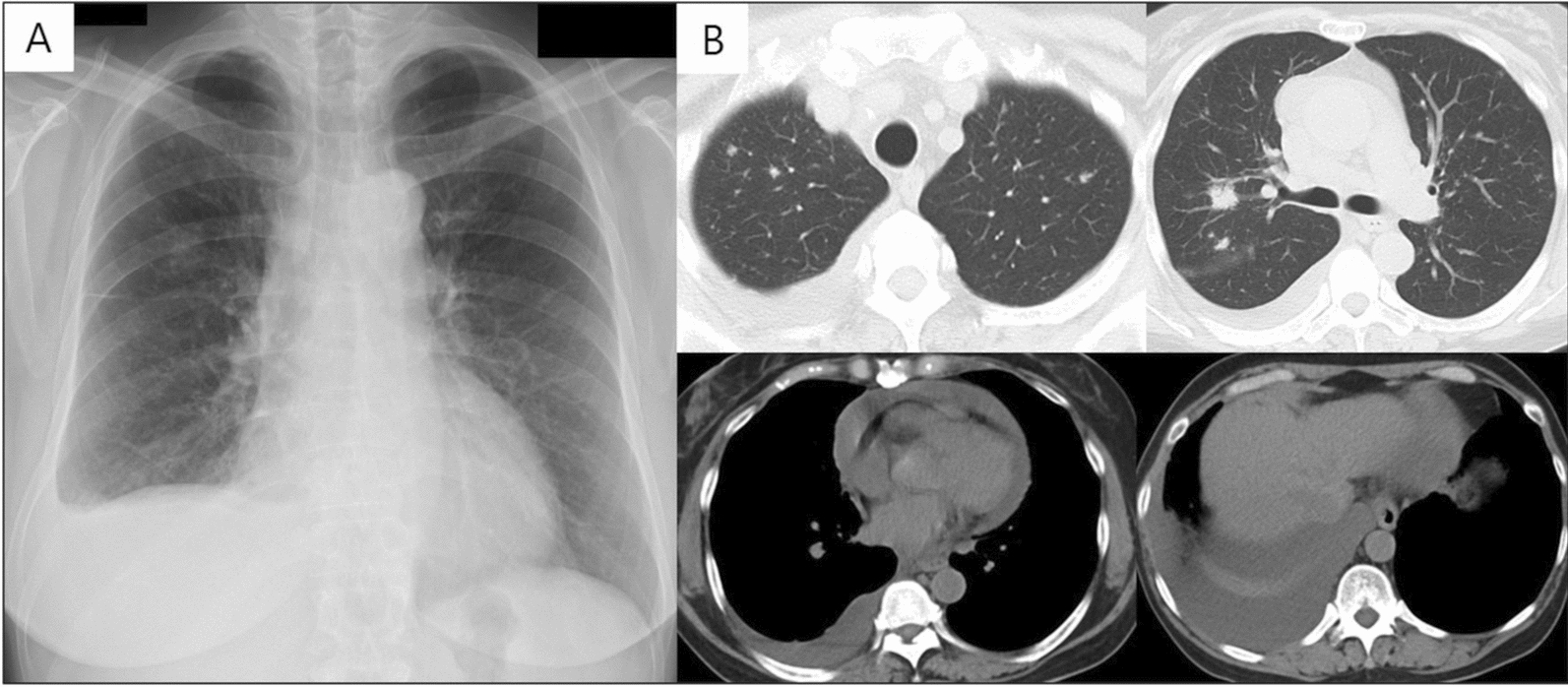
**A** Chest radiograph showed infiltrative shadows and small nodular shadows in the upper right middle lung field. The right costophrenic (CP) angle was dull. **B** A computed tomography scan showed multiple nodules in both the upper lobes and right pleural effusion and pericardial effusion

Chest radiography revealed multiple small nodular shadows in the lung fields, a right pleural effusion, and a pericardial effusion, and a chest computed tomography (CT) scan revealed multiple bilateral nodular shadows, measuring approximately 1–5 mm in diameter, with the largest nodule measuring 17 mm in the right upper lobe. Right pleural effusion and pericardial effusion were also present (Fig. 1). There was no miliary pattern, cavity lesion, lymph node enlargement, or calcification. Differential diagnoses considered at this time included bacterial pneumonia with parapneumonic effusion, congestive heart failure with pleuropericardial effusion, and collagen vascular disease. The administration of antibiotics and diuretics was ineffective. The QuantiFERON test was repeated on September 14, and was positive. The triple sputum test by Ziehl–Neelsen stain and fluorescent stain was negative, but the rapid sputum test for tuberculosis antigen performed by SRL, Inc. (Tokyo, Japan) was positive. *Mycobacterium tuberculosis* was subsequently cultured 23 days later (October 6). Unfortunately, pleural fluid could not be collected, despite performing thoracic aspiration, most likely because the amount was too small and the lesion was technically difficult to access. No significant diagnostic challenges (such as financial, or cultural) were encountered. Based on the above, the patient was diagnosed with pulmonary tuberculosis.

**Fig. 2:**
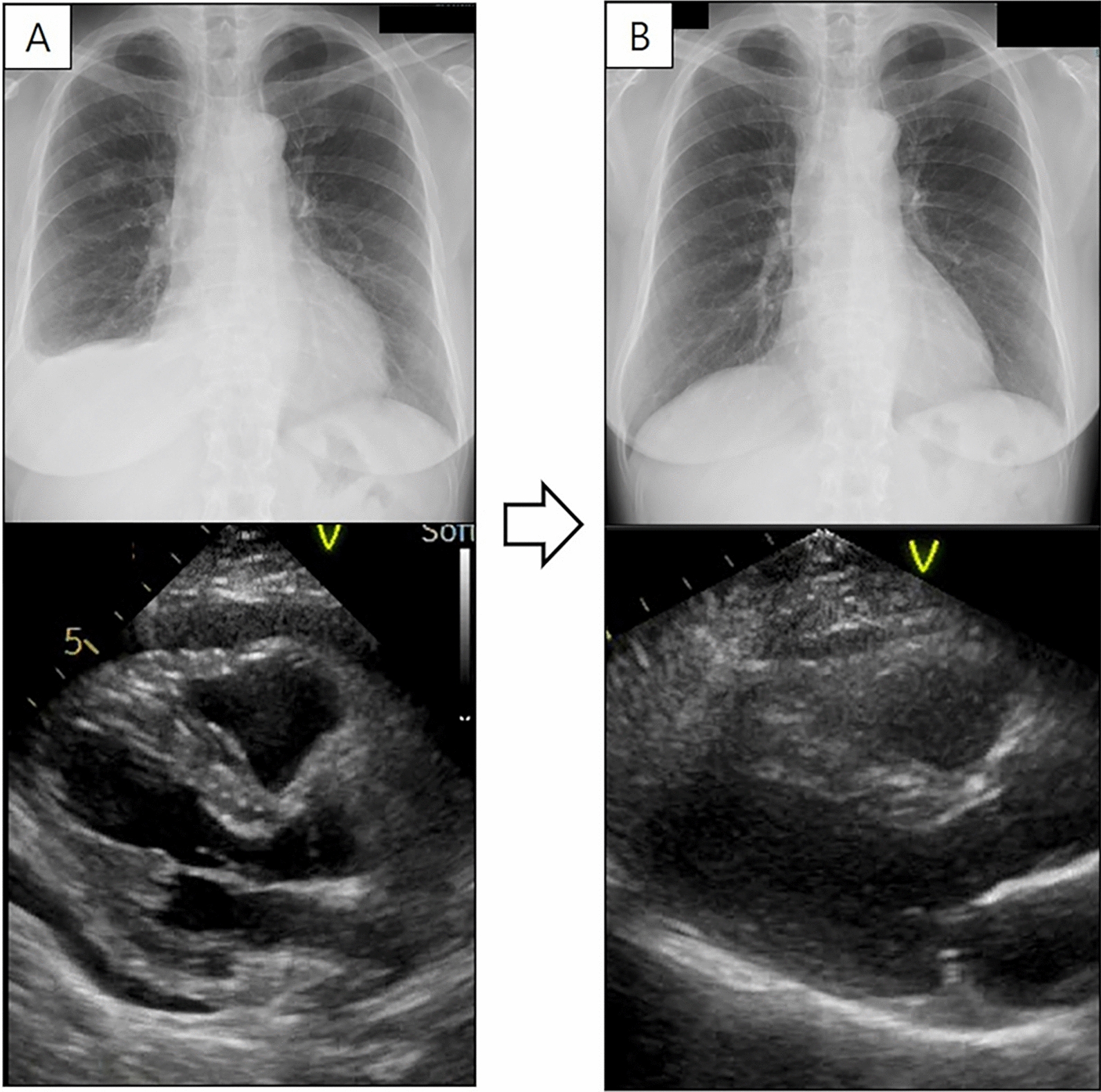
Compared with the pre-treatment (**A**), chest radiograph and cardiac echocardiography showed the pleural and pericardial effusions also completely resolved after antituberculous treatment (**B**)

**Fig. 3:**
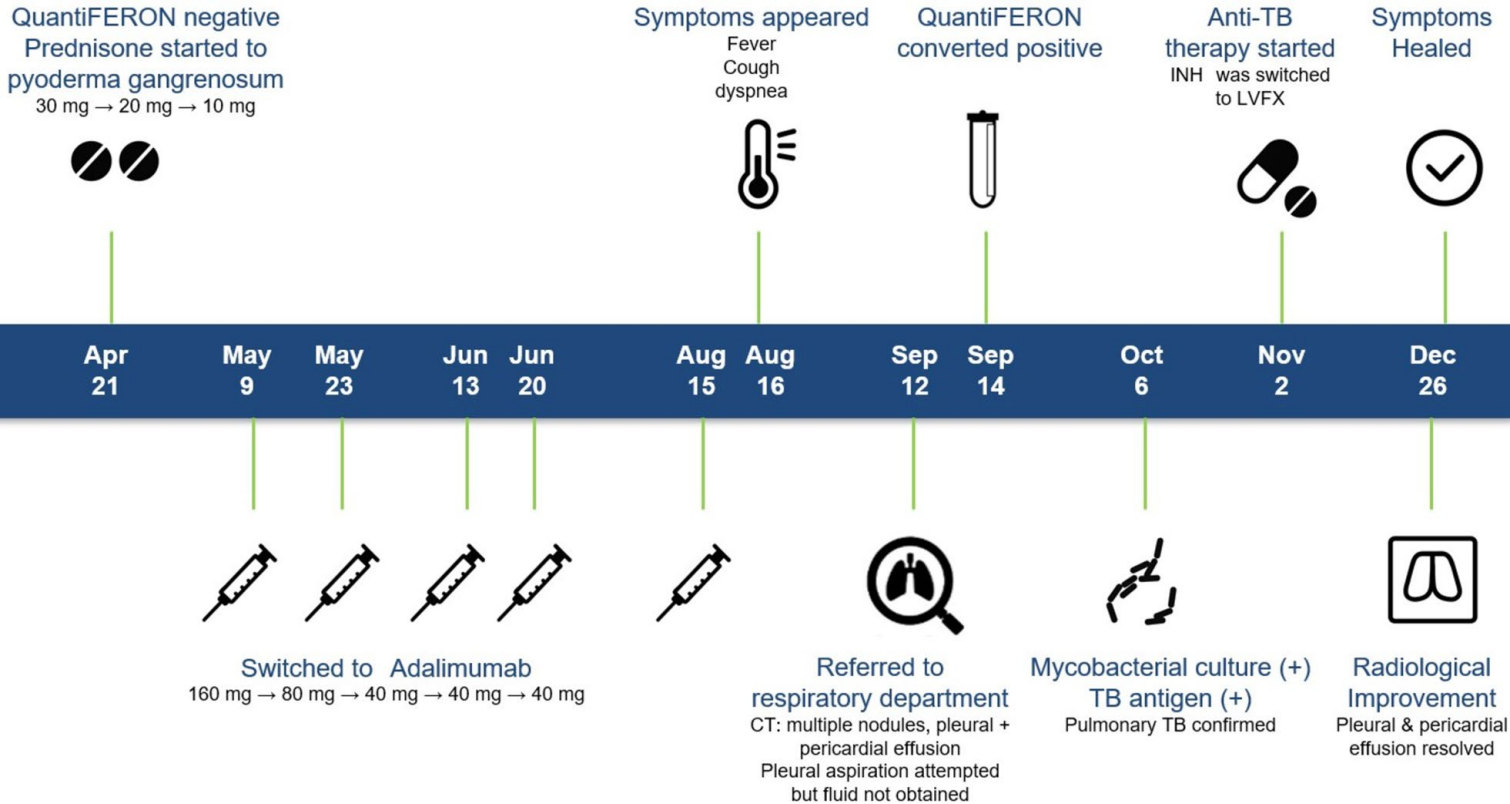
A timeline summarizing the patient's clinical course

On November 2, four-drug treatment with isoniazid, rifampicin, pyrazinamide, and ethambutol was initiated. Because drug susceptibility testing revealed resistance to isoniazid on November 4, isoniazid was changed to levofloxacin on November 5. After treatment, the patient reported progressive improvement in symptoms like fever, cough, and dyspnea. The pleural and pericardial effusions also completely resolved (Fig. 2). From the clinician’s perspective, clinical improvement was confirmed by symptom resolution and radiological improvement. Thus, we diagnosed this patient with combined tuberculous pleuropericarditis. The patient underwent four-drug combination chemotherapy for six months, followed by two-drug combination chemotherapy with rifampicin and ethambutol for three months. The patient adhered well to the anti-TB treatment regimen under directly observed treatment short course (DOTS), and no doses were missed during the treatment. There were no adverse or unanticipated events. The patient noted improvement in her symptoms and expressed general satisfaction with the result. A timeline summarizing the patient's clinical course is shown in Fig. 3. No recurrence was observed at 2 years after completion of therapy.

## Discussion

The tumor necrosis factor (TNF) plays a key role in the immune response against *Mycobacterium tuberculosis* (TB), particularly in granuloma formation and maintenance [2–4].

Adalimumab is a TNF-α inhibitor that blocks both soluble and membrane-bound TNF-α, a cytokine essential for the immune defense against TB. By inhibiting TNF-α, adalimumab disrupts the granuloma structure, allowing latent bacteria to escape and multiply, which can lead to active TB. Furthermore, adalimumab reduces macrophage function and impairs T-cell activity, including the production of interferon-gamma (IFN)-γ, thereby further increasing susceptibility to TB infection [5–9]. Furthermore, other biologic agents targeting immune pathway, such as infliximab and etanercept, have also been reported to increase the risk of TB, supporting the role of TNF-α signaling in maintaining host defense against TB. In the present case, preceding corticosteroid therapy for pyoderma gangrenosum may also have contributed to immunosuppression and increased the risk of TB development.

Examining past reported cases of extrapulmonary TB associated with anti-adalimumab, there have been a few cases of tuberculous pleuritis, pericarditis, or peritoneal effusion induced by adalimumab [10–14]. However, to the best of our knowledge, no cases of triple-combined disease, including pulmonary tuberculosis and tuberculous pleuropericarditis by adalimumab, have been reported. Therefore, this case represents a rare and clinically significant presentation of TB associated with TNF-α inhibition. This case also underscores the importance of maintaining diagnostic vigilance for rare and potentially life-threatening conditions, as highlighted in recent case reports describing unusual clinical presentations in diverse settings [15].

In our case, several differential diagnoses were considered at the time of admission, including bacterial pneumonia with parapneumonic effusion, congestive heart failure with pleuropericardial effusion, and collagen vascular diseases such as systemic lupus erythematosus. However, the administration of antibiotics and diuretics was ineffective, making bacterial pneumonia and heart failure less likely. Moreover, there was no significant increase in antibodies suggestive of collagen disease, and there were no clinical signs such as rash, arthritis, or cytopenia to suggest collagen vascular disease. Ultimately, the presence of multiple small nodular shadows in the lung fields on chest CT and a positive sputum test for *Mycobacterium tuberculosis* supported the diagnosis of pulmonary tuberculosis and tuberculous pleuropericarditis.

Pleural and pericardial involvement in this case was not microbiologically or histopathologically confirmed, as pleural fluid could not be obtained due to minimal volume and the area being technically difficult to access, which represents a limitation of this report. However, these conditions were clinically diagnosed based on characteristic imaging findings, the absence of alternative diagnoses, and the favorable response to antituberculous therapy.

**Fig. 4:**
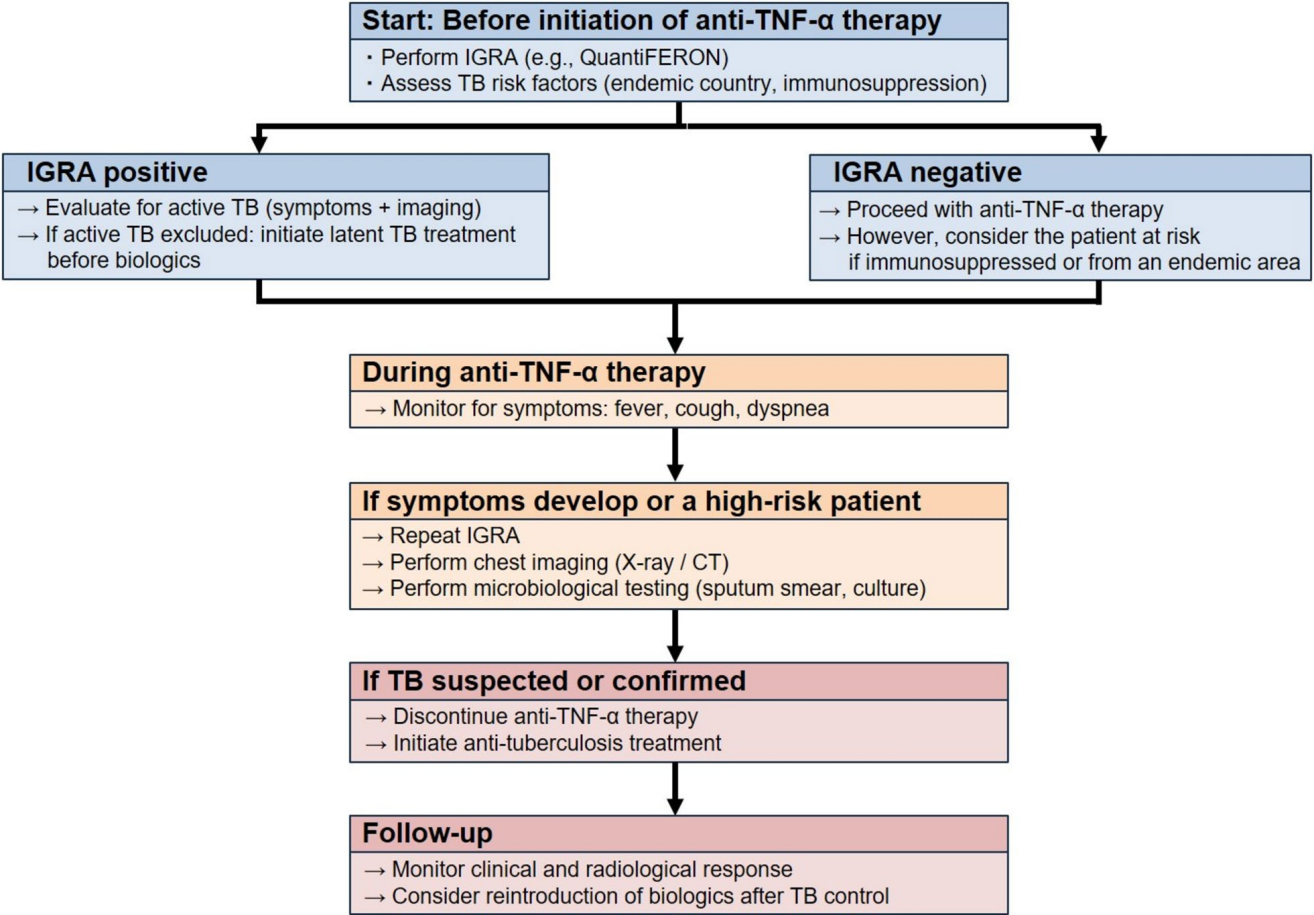
Proposed practical algorithm for TB screening and reassessment before and during anti-TNF-α therapy

IGRA is widely used for screening latent TB infection; its sensitivity may be reduced in immunocompromised patients. In the present case, the baseline IGRA was negative before adalimumab initiation but converted to positive at the time of disease onset. This finding highlights the limitation of a single baseline test and underscores the importance of repeated testing in patients receiving TNF-α inhibitors, particularly those who are symptomatic or at high risk for TB. Although microbiological culture remains the gold standard for diagnosis, its positivity rate is limited, and results may take several weeks; therefore, integration of clinical findings, imaging, and repeated immunological testing is essential for timely diagnosis [16]. Based on these considerations, a practical approach in patients receiving TNF-α inhibitors may include baseline screening with an IGRA, followed by repeat testing in the presence of symptoms or in high-risk populations, combined with prompt imaging and microbiological evaluation when TB is suspected (Fig. 4).

From an epidemiological perspective in Japan, the increase in the proportion of TB patients from Southeast Asia and the development of drug resistance are problems. In this case, the *Mycobacterium tuberculosis* isolate from a Filipino patient was resistant to isoniazid, and early modification of the treatment regimen to include levofloxacin contributed to a favorable clinical outcome.

Taken together, this case emphasizes that TB can develop during TNF-α inhibitor therapy despite negative baseline screening. Careful clinical monitoring, awareness of atypical presentations, and consideration of repeated IGRA testing are essential for early diagnosis and appropriate management, particularly in patients from endemic areas such as the Philippines.

## Conclusion

We experienced a case of pulmonary TB and pleuropericarditis following adalimumab therapy. This case emphasizes that TB can develop during TNF-α inhibitor therapy despite negative baseline IGRA results. Careful clinical monitoring, awareness of atypical presentations, and consideration of repeated IGRA testing are crucial for early detection and appropriate management, especially in high-risk individuals from endemic areas.

## Data Availability

Not applicable.

## Abbreviations

CRP: C-reactive protein
CT: Computed tomography
DOTS: Directly observed treatment short course
FDA: Food and Drug Administration
IGRA: Interferon-gamma release assay
TB: Tuberculosis
TNF-α: Tumor necrosis factor alpha
